# Biosurfactant production and hydrocarbon degradation activity of endophytic bacteria isolated from *Chelidonium majus* L.

**DOI:** 10.1186/s12934-018-1017-5

**Published:** 2018-11-03

**Authors:** Olga Marchut-Mikolajczyk, Piotr Drożdżyński, Dominika Pietrzyk, Tadeusz Antczak

**Affiliations:** 1 Institute of Technical Biochemistry, Faculty of Biotechnology and Food Sciences, Lodz University of Technology, Wólczańska 171/173, 90-924 Łódź, Poland; 2 Polytechnic Faculty, Food Technology and Human Nutrition, State University of Applied Sciences in Kalisz, Nowy Świat 4 st., 62-800 Kalisz, Poland

**Keywords:** Endophytes, Biosurfactant, Hydrocarbons, Degradation, Plant growth-promoting agent

## Abstract

**Background:**

The process of plant growth in the contaminated environment is often inhibited and entails the neutralization of harmful compounds. To reduce the negative impact of harmful compounds microorganisms produce unique compounds called biosurfactants. This paper describes the potential of culturable endophytic microorganisms from synanthropic plant-*Chelidonium majus* L. for the production of biosurfactants, as indirect plant promoting factors as well as their degradation activity. Emulsifying activity and degradation potential of tested strains were assessed by cultivation of isolates in the presence of diesel oil and waste engine oil.

**Results:**

Ten bacterial strain were isolated. Analysis of emulsifying activity revealed that all isolates possessed the ability for biosurfactant production. However, one of the isolated endophytes—2A, identified as *Bacillus pumilus*, exhibited the highest emulsifying activity (OD_500_ 1.96). The same strain has shown very high degradation potential, both for diesel oil and waste engine oil hydrocarbons. Results obtained with the Phytotoxkit tests revealed that the addition of biosurfactant isolated from *B. pumilus* 2A strain resulted in stimulation of seed germination in soil contaminated with diesel oil (137%) and waste engine oil (120%). Positive impact of the biosurfactant produced by *B. pumilus* 2A on the growth of *Sinapis alba* in hydrocarbons contaminated soil was demonstrated.

**Conclusions:**

The endophytic strain identified as *Bacillus pumilus* 2A produce biosurfactant that is able to act as plant-growth promoting agent. Endophytic bacteria isolated from *Chelidonium majus* L. exhibit potential for hydrocarbons degradation and biosurfactant production. These properties provide promising perspectives for application of biosurfactants as potential agents for bioremediation of environment contaminated with hydrocarbons.

## Background

Endophytes are microorganisms (either bacteria or fungi) inhabiting plant tissue without causing any negative physiological, epidemiological or pathogenic changes in the host organism [1, 2]. Plants can benefit from interactions with endophytes throughout the whole interaction time or only under specific conditions like biotic and abiotic stress factors [1, 2].

Endophytic species very often produce secondary metabolites which may have bioactive properties. Furthermore, these metabolites may promote plant growth and increase the resistance to stressful environmental conditions, such as the presence of xenobiotics [2–6]. Therefore, endophytes may have the ability for degradation or detoxification of organic pollutants, which makes them applicable for phytoremediation processes [7–9].

The mixture of paraffin, cycloalkanes and aromatic hydrocarbons present in the crude oil, when released into the environment, become a serious environmental problem [10]. The presence of these compounds in soil, even at low concentrations, may inhibit growth and metabolic activities of microorganisms. Furthermore, the organic pollutants may enter the food chain and, because of its carcinogenicity, toxicity and mutagenicity, they pose a serious threat to the plants, animals and humans [8].

Biosurfactants, also called as green surfactants are surface active compounds produced by microbes. These compounds are biodegradable and non-toxic, thus they do not accumulate in the environment [11–13]. Biosurfactants enhance the bioavailability of hydrophobic organic compounds, which makes them a good agent for cleaning up the environment. They may operate in one of the following manners: emulsify the non-aqueous phase liquid contaminants or increase their solubility. These features facilitate contaminants export from the solid phase and allow microorganisms adsorbed on the soil particles to access the contaminant molecule [14–16]. The increased mobility of the contamination makes it more susceptible to microbial degradation [11–13]. Biosurfactants has also a promising role in the agriculture. They take part in biofilm formation, as well as in signaling, which makes them important in plant–microbe interactions, especially in rhizosphere where they can increase the bioavailability of hydrophobic compounds for plants [17, 18]. This is particular importance for plants growing in areas contaminated with hydrocarbons. Furthermore, biosurfactants may also possess antimicrobial activity, which makes them potentially useful in elimination of plant pathogens, and also indirectly promote plant growth [18, 19]. For example, amphisin-cyclic lipopeptide, that has both antifungal and biosurfactant properties, produced by Pseudomonas fluorescens strain DSS73, prevent plants from pathogens infection, indirectly promoting plant growth [20, 21].

*Chelidonium majus* L. is a synanthropic plant occurring naturally in Asia, South America and Europe. It grows on ruderal environments, but may also grow on contaminated sites. The plant itself is toxic. However, the herb produces important bioactive compounds such as alkaloids which have antimicrobial and antiviral properties [22, 23].

Hydrocarbon degradation activity of endophytes and their ability for biosurfactant production has been recently described in the literature. However, many of the former reports pertain to the abilities of endophytic fungi to perform biodegradation, while only a few describe endophytic bacteria and their biodegradation potential or biosurfactant production ability [22–27]. We believe that this is the first report on biosurfactant production by endophytes isolated from *Chelidonium majus* L. as well as on their hydrocarbon degradation activity. That is why, the purpose of the study was twofold: to investigate the potential of culturable endophytic microorganisms isolated from *Chelidonium majus* L. plant for the production of biosurfactants, as indirect plant promoting factors and to investigate their hydrocarbon degradation activity.

## Methods

### Biological material

Endophytic bacteria were isolated from the *Chelidonium majus* L. herb. Plant samples were collected in spring 2016 from the areas of A1 motorway neighborhood, near Stryków, Poland (51°54′33.6″N 19°26′17.1″E). The plant material was carefully dug up with a spade and transported to the laboratory. Healthy parts of the plant (roots, stems, and leaves), cleaned with tap water were subjected to surface sterilization.

### Endophytes isolation

The following surface sterilization conditions were as applied: 70% ethanol—3 min, 1% sodium hypochlorite—12 min, 70% ethanol—30 s. After the final ethanol step, the plant parts were rinsed five times with a sterile water. Surface-sterile plant samples were cut with a sterile scalpel into the small pieces (~ 1 cm) under sterile conditions and placed on the sterile nutrient agar media. The efficiency of the sterilization process was verified by pipetting 100 μl of water from the last wash onto NB medium and monitoring possible microorganism growth.

In order to isolate endophytic bacteria with the ability to degrade hydrocarbons and produce biosurfactants, sterilized plant samples were placed on solid mineral medium, supplemented with 1% of diesel oil. All the plates were incubated 4 days at 30 °C. Obtained bacterial colonies were purified and stored at − 20 °C.

### The potential of the isolated endophytes for hydrocarbons degradation and biosurfactants production

Hydrocarbon degradation activity of isolated endophytes and their ability for biosurfactant production were assessed in liquid culture conditions. Bacterial endophytes were cultivated in mineral liquid medium, which contained [g L^−1^]: 0.7 KCl, 2.0 KH_2_PO_4_, 3.0 Na_2_HPO_4_, 1.0 NH_4_NO_3_, and trace element solution (4.0 MgSO_4_, 0.2 FeSO_4_, 0.2 MnCl_2_, and 0.2 CaCl_2_) [28]. Mineral medium was supplemented with diesel oil (5% v/v) or waste engine oil (5% v/v) as a sole carbon source (culture conditions: 10 days, 30 °C, 160 rpm). For each of the endophytic strains, the hydrocarbon degradation activity analysis was performed in triplicate.

### Hydrocarbon degradation activity

After the separation of bacterial biomass from the culture medium (centrifugation: 18,000 rpm, 4 °C, 20 min, Hermle LaborTechnik Z36HK centrifuge), the organic phase (non-degraded hydrocarbons) from the upper layer of the supernatant was collected. The residual hydrocarbons fraction was diluted 1:1 in hexane, and subjected to GC analysis. The results were compared to those obtained for control samples, containing non-inoculated mineral medium with the addition of diesel oil (5% v/v) or waste engine oil (5% v/v).

### Gas chromatography

Hewlett–Packard 5890 GC chromatograph, with the flame ionization detector (FID), was used for the determination of diesel oil and waste engine oil hydrocarbons biodegradation. The GC analysis conditions were: DB-1 column (30 m, 0.53 mm i.d., 1.0 μm film thickness); helium carrier gas (flow rate of 2 ml/min); temperature program: Oven: from 60 to 260 °C at a rate of 4 °C/min; split/splitless injector and detector (FID): 260 °C; injection volume: 1 μl; internal standard: *o*-terphenyl (10 mg/ml).

### Emulsifying activity and Emulsion index (E24)

The emulsifying activity and emulsion index (E24) were determined using the Pearce and Kinsella method [29]. The reaction mixture for the emulsifying activity assay contained 3 ml of the culture supernatant (culture conditions same as in “The potential of the isolated endophytes for hydrocarbons degradation and biosurfactants production” section), 1 ml of diesel oil and 1 ml of 0.1 M phosphate buffer (pH 7). The mixture was homogenized for 30 s at 18,000 rpm in Yellow Line DI 18 Basic homogenizer and immediately after, 0.1 ml of the homogenized mixture was transferred to 1 ml of 0.1% sodium dodecyl sulphate (SDS). Subsequently, the absorbance at 500 nm wavelength against water as a control sample was measured on the UV/VIS T80+ spectrophotometer.

Subsequently, the stability of the biosurfactant and its ability to emulsify liquid hydrocarbons (diesel oil, waste engine oil) were examined. Each test tube contained 2 ml of the cell—free broth with the biosurfactant and 2 ml of the pollutant. Such prepared mixture was vortexed vigorously for 2 min and left for 24 h. The emulsion index (E24) was calculated according to the equation: E24 = height of the emulsion layer/total height of liquid × 100.

### Species identification of the endophytic strain 2A

Endophytic strain 2A, for which the highest emulsifying activity has been obtained, was identified through 16S rRNA sequencing. For the purpose of genomic DNA isolation, a bacterial biomass obtained from 24 h bacterial culture was used. EurX GeneMATRIX Tissue and Bacterial DNA purification Kit was applied [30]. 16S rDNA (ribosomal DNA) was amplified with the use of universal primers called Golden Mixture 7 [31]. The PCR mixture consisted: 2 μl of DNA template, 10 μl of GoTaq Flexi Buffer, 1 μl of 10 mM l-1 of PCR Nucleotide mix, 2 μl of 25 mM l-1 MgCl2, 0.5 μl of 0.1 μM l-1 each of the reverse and forward primers, 0.25 μl of 95 U/μl GoTaq G2 Hot Start Polymerase in 50 μl of the final reaction volume. The PCR reaction conditions were: denaturation at 95 °C for 2.25 min, 35 cycles: 94 °C for 1.25 min, 48 °C for 0.5 min, 58 °C for 0.75 min, 72 °C for 1.25 min, followed by final extension of 10 min at 72 °C.

The first PCR with Golden Mixture 7 primers was performed in order to identify the primer pair amplifying 16S rDNA sequence of 2A bacterial strain. This way the combination of forward Fn6 (5′ CCAGCAGCCGCGGTAATAC 3′) and reverse Rn3 (5′ GGCGTGGACTACCAGGGTATC-3′) primers were picked for the second, 16S rDNA specific amplification [31]. Both experiments were visualized by electrophoresis in a 2.5% agarose gel with use of Midori Green DNA Stain. Final PCR product corresponding to 16S rDNA sequence of the strain 2A was sent for sequencing to Genomed (Warsaw, Poland). The obtained sequence was aligned against nucleotide sequences available in the NCBI database using BLAST.

### Hydrocarbon degradation activity of *B. pumilus* 2A strain

The potential of *B. pumilus* 2 A endophytic bacteria for hydrocarbon degradation was evaluated in mineral medium containing 5% of diesel oil or waste engine oil. Evaluation residual hydrocarbons after biodegradation was performed as described above for all endophytes isolated from *Chelidonium majus* L. GC analysis were conducted as mentioned above in section of Gas chromatography. Obtained chromatograms were quantified with respect to Alkane standard mixture (Sigma-Aldrich) and Aromatic hydrocarbon standard (Sigma-Aldrich).

### Isolation of biosurfactant produced by *B. pumilus* 2A

The biosurfactant extraction was based on centrifugation of culture liquids at 18,000 rpm for 15 min at 4 °C. Subsequently, bacterial cell-free culture medium was acidified with 6 M hydrochloric acid to pH 2 and left overnight at 4 °C. After this time the liquid was centrifuged once more at conditions as above. The supernatant was removed, while the precipitate was dissolved in 0.1 M NaHCO_3_ and extracted with chloroform and methanol with ratio of 2:1. The organic phase, containing the biosurfactant, was collected and the solvents were evaporated in a vacuum evaporator [32].

After the extraction initial chemical analysis of biosurfactant was performed. The presence of carbohydrate group has been found on the basis of the sugars determination with the phenol and sulfuric acid method [33]. 1 ml of biosurfactant solution was added to 0.5 ml of 5% phenol solution and 2.5 ml of concentrated sulfuric acid was added. The samples were left for 30 min at room temperature and then the absorbance of the samples was measured at 490 nm against a control sample containing water instead of biosurfactant solution. Protein content was determined using the Bradford method [34]. 1 ml of the biosurfactant solution was added to 1 ml of 1 M NaOH and then incubated in boiling water for 10 min. After this time, 50 μl of the mixture was added to 1.5 ml of Bradford reagent and the absorbance at 590 nm was measured against the control sample containing water instead of the biosurfactant solution. Also, the presence of carbohydrates, proteins and lipids was examined using the anthrone, ninhydrin and saponification tests [35, 36]. Lipids content was assayed by thin layer chromatography. The biosurfactant solution was extracted with 20 ml of chloroform and then subjected to TLC analysis.

### Plant growth promoting ability analysis

Plant growth promoting facility of biosurfactant obtained from *B. pumilus* 2 A endophytic strain was examined in soil contaminated with diesel oil or with waste engine oil, using Phytotoxkit test. During bioassays the inhibition, presence and increase in seeds germination after 3 days of the exposure of seeds to diesel oil or waste engine oil (5% w/w) in soil with or without the addition of biosurfactant were measured. The Phytotoxkit tests (MicroBioTests Inc.) were carried out in accordance with standard procedure of this assay [37], using seeds of *Sorghum saccharatum*, *Lepidium sativum* and *Sinapis alba*. The concentration of biosurfactant was 10 mg/g.

### Data analysis

The R program (version 3.2.2) for Windows was used for data analysis, which were represented as the mean ± standard deviation (SD) of the quintuples samples. For the hydrocarbon degradation activity, emulsifying activity and emulsion index (E24), the significance of differences between means was estimated by one-way ANOVA, and post hoc Tuckey test.

## Results and discussion

### Isolation of endophytic bacteria capable of hydrocarbons degradation

The reports concerning the isolation of endophytic bacteria from the medicinal plants focus mainly on the ones growing in Asia and South America [38–41]. The literature data on endophytes isolated from European medicinal plants are still scarce [42–46]. Goryluk et al. isolated endophytic bacteria from *Chelidonium majus* L. herb and investigated their biological activity [42, 46]. However, the ability of endophytes from *C. majus* L. for biosurfactant production and hydrocarbons degradation have not been studied.

Plants used in the present study grew in the environment where they were exposed to the contamination with the products of fuel combustion and volatile hydrocarbons. Therefore, there is the increased possibility of finding endophytic microorganisms with the potential for hydrocarbons degradation.

Table 1 represents the symbols of all isolated endophytic bacteria, together with the parts of the plant from which they were isolated. For the purpose of the identification of endophytes with the ability to degrade hydrocarbons, microorganisms were cultured on NB medium containing 1% of diesel oil. Six out of 10 isolated strains (2A, 4B, EN9, EN10, EN1, EN18) have exhibited growth both on contaminated and uncontaminated medium. These were considered as possible petroleum degraders. For the rest of the isolates, very little or no growth was observed on the contaminated medium. The effectiveness of surface sterilization method was confirmed by the lack of microbial growth in the control sample after 10 days of incubation.

**Table 1:** Endophytic bacteria isolated from different parts of *Chelidonium majus* L.

Bacteria	
Endophyte symbol	Part of plant were isolated from
EN1	Root
2A	Leaf
EN10	Leaf
EN3	Root
EN11	Rhizosphere
4B	Rhizosphere
EN5	Leaf
EN6	Leaf
EN18	Root
EN9	Root

### Hydrocarbon degradation activity

The ability of isolates for hydrocarbons degradation was tested in the liquid mineral medium supplemented with 5% (v/v) of diesel or waste engine oil, as a sole carbon source. The efficiency of hydrocarbons degradation varied, depending on the tested endophyte and hydrocarbons source. The hydrocarbons depletion observed for the given endophytic strain is presented in Table 2.

**Table 2:** Hydrocarbon degradation activity of isolated endophytic microorganisms (mean ± SD, n = 3)

Endophyte symbol	Hydrocarbon loss (diesel oil) [%]	Hydrocarbon loss (waste engine oil) [%]
2A	98.02 ± 4.90	92.81 ± 4.64
4B	95.48 ± 5.54	62.98 ± 3.14
EN9	94.58 ± 4.92	55.50 ± 2.77
EN10	93.12 ± 5.03	51.39 ± 2.76
EN11	87.91 ± 4.39	57.33 ± 2.86
EN1	95.47 ± 4.77	75.24 ± 3.76
EN6	76.37 ± 3.82	47.69 ± 2.38
EN18	91.39 ± 5.12	48.03 ± 2.40
EN5	68.47 ± 3.42	41.77 ± 2.43
EN3	71.25 ± 3.56	38.79 ± 1.93
Control Sample	23.33 ± 1.16	24.70 ± 1.2

Hydrocarbon degradation activity of endophytes varied from 9 to 92.81% and 24 to 75.9% for diesel oil and waste engine oil, respectively. For diesel oil hydrocarbons only 5 of tested endophytes resulted in a significant difference in their hydrocarbon degradation activity. However, in case of waste engine oil degradation statistical differences were observed for all tested endophytes. All strains were significantly different from the Control Sample.

Pawlik et al. examined the potential for hydrocarbons degradation (diesel oil, *n*-hexane and *p*-xylene degradation) of endophytic bacteria isolated from *Lotus corniculatus* and *Oenothera biennis* growing in contaminated soil [47]. Isolated endophytes belonged mainly to the genera of *Pseudomonas, Stenotrophomonas, Rhizobium,* and *Rhodococcus*. Over 90% of them were able to utilize diesel oil as a carbon source [47]. Also, Philips et al. studied the degradation abilities of endophytes isolated from the prairie plants. In his study, the degradation potential of isolated strains did not exceed 60% [48]. Baoune et al. isolated endophytic *Streptomyces* spp. with extremely high—98% efficiency of petroleum hydrocarbons removal [49].

Although the use of endophytic microorganisms for petroleum compounds degradation has been previously reported [47, 49, 50], here for the first time the potential of bacterial endophytes isolated from the synanthropic plant—*C. majus* L. for degradation of such compounds was shown.

### Emulsifying activity and Emulsion index (E24)

Because of the hydrophobic nature of petroleum compounds, the ability of microorganisms to synthesize biosurfactants, that can enhance the availability of the pollutants is crucial [49]. The ability of isolated endophytic microorganisms for biosurfactants production was tested by measuring the emulsifying activity and emulsion index (E24) of the post-culture medium. The emulsifying activity scores are summarized in Table 3. In case of diesel oil statistical differences in emulsifying activity were observed for all ten endophytes. On the other hand, for waste engine oil only for two endophytes (EN5, EN1) no statistical differences were noted. Four out of ten isolates differ significantly from the rest of the endophytes and show high emulsifying activity. However, the 2A strain turned out to be the most effective biosurfactant producer. The values of the emulsifying activity for this strain reached 1.96 and 1.2 for diesel and waste engine oil, respectively.

**Table 3:** Emulsifying activity of isolated endophytic microorganisms (mean ± SD, n = 3)

Emulsifying activity of isolated endophytic bacteria		
Endophyte symbol	Diesel oil	Waste engine oil
EN5	0.89 ± 0.04	0.81 ± 0.16
EN3	0.81 ± 0.04	0.71 ± 0.33
2A	1.96 ± 0.09	1.2 ± 0.19
4B	0.30 ± 0.01	0.38 ± 0.13
EN9	0.23 ± 0.01	0.63 ± 0.07
EN10	0.53 ± 0.02	0.77 ± 0.002
EN11	0.43 ± 0.02	0.57 ± 0.02
EN1	1.15 ± 0.05	0.82 ± 0.02
EN6	1.71 ± 0.09	0.89 ± 0.12
EN18	1.78 ± 0.09	0.96 ± 0.07

The values of the emulsion index varied from 29 to 47% for diesel oil and from 23 to 65% for waste engine oil as presented in Table 4. The most stable emulsion according to the E24 index was obtained for 2A strain (E24 = 65%), cultivated on the media contaminated with waste engine oil. This is a significantly higher result than what Baoune et al. observed for the endophytic *Streptomyces* spp., isolated from a plant growing in Algeria, in the case of which E24 did not exceed 46% [49]. For 3rd, 7th, 10th, and 14th day of diesel oil biodegradation there were statistical differences in E24 values between tested microorganisms. However, for waste engine oil no statistical differences were observed in the 3rd day of biodegradation between EN1 and EN18 as well as between 2A and EN6. In 7th, 10th and 14th day of the process statistical differences between tested endophytes were observed.

**Table 4:** Emulsifying index IE 24 during biodegradation process

Endophyte symbol	Carbon source	Biodegradation (day)			
		3	7	10	14
		Emulsifying index E24 (%)			
EN1	Diesel oil	12.41 ± 1.98	24.18 ± 3.17	25.03 ± 3.01	29.33 ± 1.49
	Waste engine oil	28.82 ± 1.26	37.66 ± 2.44	41.80 ± 1.72	45.51 ± 2.64
2A	Diesel oil	10.27 ± 1.04	21.39 ± 0.74	35.75 ± 7.16	37.25 ± 3.16
	Waste engine oil	19.90 ± 1.30	44.33 ± 3.26	65.29 ± 3.52	65.58 ± 0.72
EN6	Diesel oil	15.25 ± 0.76	23.05 ± 1.30	37.13 ± 5.95	40.13 ± 2.87
	Waste engine oil	20.50 ± 1.70	29.39 ± 2.50	26.39 ± 3.81	23.12 ± 2.36
EN18	Diesel oil	17.41 ± 3.27	45.91 ± 4.96	45.91 ± 4.96	47.11 ± 3.86
	Waste engine oil	29.44 ± 2.47	31.06 ± 2.93	31.06 ± 2.93	32.01 ± 1.27

### Results of the taxonomy determination of 2A strain

The taxonomy of the endophytic 2A strain has been identified by phylogenetic analysis of 16S rDNA sequence. For 2A strain high sequence identity to *Bacillus pumilus* FJAT-44675 16s rDNA (99.7%) was observed. Also, analysis of biochemical and physiological properties of 2A strain, revealed close similarity to *B. pumilus*.

There are only a few reports on *Chelidonium majus* L. bacterial endophytes. Goryluk et al. isolated 34 bacterial endophyte strains. Most of the isolates belonged to the Bacillus genera and was identified as *B. licheniformis, B. subtilis, B. thuringiensis, B. cereus* and *B. amyloliquefaciens*. None of the isolates belonged to the genera of *Bacillus pumilus* [42]. However Kumar et al. in their report on bacterial endophytes of *Curcuma longa* L. isolated endophyte from six different species, including *B. pumilus*. Endophytic *B. pumilus* strains isolated from the herbal plants such as *Ocimum sanctum* and *Curcuma longa* L. showed the ability for production of plant growth promoting substances [51–53]. However, none of the authors investigated degradation ability and biosurfactant production by the isolated endophytes.

### Hydrocarbon degradation activity of endophytic *Bacillus pumilus* 2A strain

Mineral medium enriched with 5% of diesel oil or waste engine oil was used to evaluate the ability of endophytic *Bacillus pumilus* 2A, to degrade hydrocarbons, after 10 days of cultivation. Endophytic *Bacillus pumilus* 2A demonstrated the n-alkanes and aromatic hydrocarbons degradation ability (Table 5). The most efficient degradation was observed for n-alkanes from C9 to C14, for both diesel oil and waste engine oil. Interestingly, the strain showed also the ability to degrade benzene, although the efficiency of simple aromatic compound degradation was lower than for aliphatic hydrocarbons.

**Table 5:** Concentration of selected hydrocarbons in diesel oil and waste engine oil and their degradation by endophytic *B. pumilus* 2A

Hydrocarbon	Concentration (ppm)		Hydrocarbons degradation (%)	
	Diesel oil	Waste engine oil	Diesel oil	Waste engine oil
Nonane	460.8 ± 13.8	230.5 ± 6.9	98.5 ± 2.9	75.3 ± 2.2
Decane	453 ± 13.6	307.5 ± 9.2	99.9 ± 2.9	81.2 ± 2.5
Undecane	504.7 ± 15.1	356 ± 10.7	83.9 ± 2.5	92.5 ± 2.8
Dodecane	583.5 ± 17.5	408.2 ± 12.2	94.5 ± 2.8	75.6 ± 2.3
Tridecane	605.1 ± 18.2	504.3 ± 15.1	77 ± 1.7	72 ± 1.9
Tetradecane	14.1 ± 0.4	12.5 ± 0.4	89 ± 2.7	79.5 ± 2.1
Heptadecane	501.4 ± 15.0	564.3 ± 16.9	49 ± 1.5	39 ± 1.2
Eicosane	305.8 ± 9.2	426.8 ± 12.8	76 ± 2.3	63.5 ± 1.9
Benzene	2.05 ± 0.06	2.95 ± 0.09	76.5 ± 2.3	66.9 ± 2.0
Toluene	0.98 ± 0.03	1.64 ± 0.05	67.5 ± 2.0	56.5 ± 1.7
Pristane	471 ± 14.1	523.5 ± 15.7	59.2 ± 1.8	46.8 ± 1.4

The reports concerning bacterial strains capable of both aliphatic and aromatic hydrocarbons degradation are scarce [22]. Buzanello et al. described *B. pumilus* strain that could degrade dibenzothiophene and its derivative metabolites [54]. Also Surendra et al. found that, catechol 1,2 dioxygenase (C12D) from *B. pumilus* MVSV3 can be very efficient in the removal of BTEX from the environment [55]. However, none of these strains were isolated from the plant material. Baoune et al. described endophytic *Streptomyces* spp. with the ability to degrade a broad profile of diesel oil hydrocarbons. However, the strain was not able to metabolize benzene [49].

The potential of endophytic *Bacillus pumilus* 2A strain for hydrocarbons biodegradation may be related to the fact that *Chelidonium majus* L. plant from which endophytic bacteria were isolated, grew in the environment exposed to the hydrocarbons contamination [49].

### Plant growth promoting effect of biosurfactant isolated from endophytic *B. pumilus* 2A strain in hydrocarbons contaminated soil

**Fig. 1:**
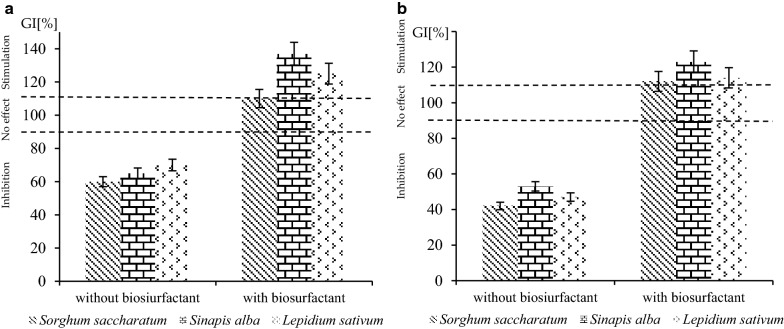
Germination index values (% of control) for soil contaminated with **a** diesel oil and **b** waste engine oil with or without the addition of biosurfactant from endophytic *B. pumilus* 2A

The presence of contaminants in soil, e.g. hydrocarbons may inhibit or even indispose plant growth. Biosurfactants, which facilitates contaminants export from the solid phase and allows microorganisms adsorbed on the soil particles to access the contaminant molecule may indirectly promote plant growth, by increasing the bioavailability of hydrophobic compounds for microorganisms inhabiting rhizosphere and plants [14–16]. In this study, the impact of biosurfactant isolated from endophytic *B. pumilus* 2A on germination and seedling growth of three plant species in soil contaminated with hydrocarbons was evaluated (Fig. 1). Initial studies on chemical structure of the biosurfactant were performed. Results of Bradford test as well as ninhydrin test show the absence of protein or amino acids in examined biosurfactant. The formation of green color in anthrone test as well as results obtained using Dubois method denoted the presence of carbohydrates in the biosurfactant. Saponification test revealed the presence of lipids in biosurfactant, which was also analysed using TLC method. Thus, after biochemical analysis it can be assumed that the biosurfactant produced by 2A strain is glycolipid. For all tested plants the stronger response to the presence of biosurfactant in soil was obtained for *Sinapis* alba. Both, in soil contaminated with diesel oil and waste engine oil a stimulation of seeds germination was observed (137% and 120% respectively, as compared to the control). Also, a stimulation in germination of *Lepidium sativum* seeds has been observed in soil contaminated with diesel oil, in the presence of biosurfactant. For *Sorghum saccharatum* the weakest response to the presence of biosurfactant in soil was noted. To the best of our knowledge, there is no study on the use of biosurfactants isolated from endophytic microorganisms as plant growth promoting agents. However, further research are needed to investigate the influence of different concentration of biosurfactant on plant growth enhancement and the mechanism of observed phenomenon.

## Conclusions

In conclusion, endophytic bacteria isolated from *Chelidonium majus* L. exhibit potential for hydrocarbons degradation and biosurfactant production. These properties may provide promising perspectives for their application as potential agents for bioremediation of a hydrocarbons contaminated environment. Furthermore, plant-growth promoting ability of the biosurfactant may be use to promote plant growth in hydrocarbon contaminated sites. The endophytic strain identified as *Bacillus pumilus* 2A produce biosurfactant that is able to act as plant-growth promoting agent. However, the precise knowledge of the mechanism of observed phenomenon will be important in evaluating possibility of application biosurfactants to promote plant growth, especially in contaminated areas.

## References

[1] Malfanova NV. Endophytic bacteria with plant growth promoting and biocontrol abilities. Ph.D. thesis, 2013, Leiden: Leiden University.

[2] Pisarska K, Pietr SJ (2014). Bakterie endofityczne–ich pochodzenie i interakcje z roślinami. Post Mikrobiol..

[3] Rosenblueth M, Martinez-Romero E (2006). Bacterial endophytes and their interactions with hosts. Mol Plant-Microbe Interact J.

[4] Schulz B, Boyle C (2006). What are Endophytes? Microbial root endophytes.

[5] Hardoim PR, Overbeek LS, Elsas JD (2008). Properties of bacterial endophytes and their proposed role in plant growth. Trends Microbiol.

[6] Mishra A, Singh SP, Mahfooz S, Bhattacharya A, Mishra N, Shirke PA, Nautiyal CS (2018). Bacterial endophytes modulates the withanolide biosynthetic pathway and physiological performance in *Withania somnifera* under biotic stress. Microbiol Res.

[7] Nair DN, Padmavathy S (2014). Impact of endophytic microorganisms on plants, environment and humans. Sci World J..

[8] Afzal M, Khan QM, Sessitsch A (2014). Endophytic bacteria: prospects and applications for the phytoremediation of organic pollutants. Chemosphere.

[9] Zahoor M, Irshad M, Rahman H, Qasim M, Afridi SG, Qadir M, Hussain A (2017). Alleviation of heavy metal toxicity and phytostimulation of *Brassica campestris* L. by endophytic *Mucor* sp. MHR-7. Ecotoxicol Environ Safety..

[10] Rabęda I, Woźny A, Krzesłowska M. Bakterie i Grzyby Mikoryzowe Zwiększają Wydajność Roślin w Fitoremediacji Metali Śladowych. KOSMOS: Problemy Nauk Biologicznych. 2011;60:423–433, in polish.

[11] Saharan BS, Sahu RK, Sharma D (2011). A review on biosurfactants: fermentation, current developments and perspectives. Genetic Eng Biotechnol J.

[12] Chakrabarti S. Bacterial biosurfactant: characterization, antimicrobial and metal remediation properties. 2012. (Doctoral dissertation).

[13] Sharma D (2016). Biosurfactants in Food, Part of the Springer Briefs in Food, Health, and Nutrition book series (BRIEFSFOOD).

[14] Bustamante M, Durán N, Diez MC (2012). Biosurfactants are useful tools for the bioremediation of contaminated soil: a review. J Soil Sci Nutr.

[15] Khan MSA, Singh B, Cameotra SS (2015). Biological applications of biosurfactants and strategies to potentiate commercial production biosurfactants. Prod Util Processes Technol Econ..

[16] Hausmann R, Syldatk C (2015). Types and classification of microbial surfactants, biosurfactants. Prod Util Processes Technol Econ..

[17] Ron EZ, Rosenberg E (2011). Natural roles in biosurfactants. Environ Microbiol.

[18] Sachdev DP, Cameotra SS (2013). Biosurfactants in agriculture. Appl Microbiol Biotechnol..

[19] Liu WW, Yin R, Lin XG, Zhang J, Chen XM, Li XZ, Yang T (2010). Interaction of biosurfactant-microorganism to enhance phytoremediation of aged polycyclic aromatic hydrocarbons (PAHS) contaminated soils with alfalfa (*Medicago sativa* L.). Huan Jing Ke Xue..

[20] Andersen JB, Koch B, Nielsen TH, Sorensen D, Hansen M, Nybroe O (2003). Surface motility in *Pseudomonas* sp. DSS73 is required for efficient biological containment of the root-pathogenic microfungi *Rhizoctonia solani* and *Pythium ultimum*. Microbiology..

[21] Saraf M, Pandya U, Thakkar A (2014). Role of allelochemicals in plant growth promoting rhizobacteria for biocontrol of phytopathogens. Microbiol Res.

[22] Zhang X, Liu X, Wang Q, Chen X, Li H, Wei J, Xu G (2014). Diesel degradation and potential of endophytic bacteria isolated from Scirpus triqueter. Int Biodeter Biodegr..

[23] Singh M, Kumar A, Singh R, Pandey KD (2017). Endophytic bacteria: a new source of bioactive compounds. 3 Biotech..

[24] Da Silva MET, Nascimento CC, Junior SD, Albuquerque PM (2014). Biosurfactant production by *Myrciaguianensis* endophytic fungi. BMC Proc.

[25] Singh MJ, Sedhuraman P (2015). Biosurfactant, polyethene, plastic, and diesel biodegradation activity of endophytic *Nocardiopsis* sp. mrinalini9 isolated from *Hibiscus rosasinensis* leaves. Bioresour Bioproc..

[26] Lima JMS, Pereira JO, Batista IH, de Queiroz CN, dos Santos JC, de Araújo SP, Pantoja MC, da Mota AJ, de Azevedo JL (2016). Potential biosurfactant producing endophytic and epiphytic fungi, isolated from macrophytes in the Negro River in Manaus, Amazonas, Brazilc. Afr J Biotechnol..

[27] Shalini DS, Bhaskara KV (2017). Exploration of antimicrobial compounds from *Streptomyces* S9 against phytopathogen, *Corynespora cassicola*. (Berk & Curtis) J Biopest..

[28] Richard JY, Vogel TM (1999). Characterization of a soil bacterial consortium capable of degrading diesel fuel. Int Biodeterior Biodegr..

[29] Pearce KN, Kinsela JE (1978). Emulsifying properties of proteins: evaluation of a turbidimetric. Tech J Agric Food Chem.

[30] GeneMATRIX Tissue and Bacterial DNA Purification Kit, Version 1.0. 2009. https://www.bmlabosis.com/uploads/eb6e252f357741f0ac1c05920ad36e45.pdf.

[31] Barghouthi SA (2011). A universal method for the identification of bacteria based on general PCR primers. Ind J Microbiol.

[32] Sumiardi A, Mangunwardoyo W, Hudiyono S, Susilaningsih D (2012). Biosurfactant characterization of bacterial consortium from soil contaminated hydrocarbon in Cepu Area, Cetral Javam Indonesia. Int J Sci Res Publ.

[33] Dubois M, Gilles EKA, Hamilton JK, Rebers PA, Smith F (1956). Calorimetric dubois method for determination of sugar and related substances. Anal Chem.

[34] Bradford MM (1976). A rapid and sensitive method for quantitation of microgram quantities of protein utilizing the principle of protein-dye binding. Anal Biochem.

[35] Patowary K, Saikia RR, Kalita MC, Deka S (2014). Degradation of polyaromatic hydrocarbons employing biosurfactant-producing *Bacillus pumilus* KS2. Ann Microbiol.

[36] Patowary K, Patowary R, Kalita MC, Deka S (2017). Characterization of biosurfactant produced during degradation of hydrocarbons using crude oil as sole source of carbon. Front Microbiol.

[37] Phytotoxkit Seed germination and early growth microbiotest with higher plants, standard operational procedure. MicroBio Tests. 2015. http://www.microbiotests.be/toxkit-microbiotests/test-protocols/.

[38] Janardhan BJ, Vijayan K (2012). Types of endophytic bacteria associated with traditional medicinal plant *Lantana camara* Linn. Pharmacogn J..

[39] Alvin A, Miller KI, Neilan BA (2014). Exploring the potential of endophytes from medicinal plants as sources of antimycobacterial compounds. Microbiol Res.

[40] Beiranvand M, Amin M, Hashemi-Shahraki A, Romani B, Yaghoubi S, Sadeghi P (2017). Antimicrobial activity of endophytic bacterial populations isolated from medical plants of Iran. Iran J Microbiol..

[41] Khan AL, Gilani SA, Waqas M, Al-Hosni K, Al-Khiziri S, Kim Y-H, Al-Harrasi A (2017). Endophytes from medicinal plants and their potential for producing indole acetic acid, improving seed germination and mitigating oxidative stress. J Zhejiang Univ Sci B.

[42] Goryluk A, Rekosz-Burlaga H, Błaszczyk M (2009). Isolation and characterization of bacterial endophytes of Chelidonium majus L. Pol J Microbiol..

[43] Machavariani NG, Ivankova TD, Sineva ON, Terekhova LP (2014). Isolation of endophytic *Actinomycetes* from medicinal plants of the Moscow Region, Russia. World Appl Sci J.

[44] Golinska P, Wypij M, Agarkar G, Rathod D, Dahm H, Rai M (2015). Endophytic actinobacteria of medicinal plants: diversity and bioactivity. Antonie Van Leeuwen.

[45] Rekosz-Burlaga H, Tokarczyk E, Szczepaniak J, Goryluk-Salmonowicz A (2016). Antagonistic activity of plant-associated microorganisms against *Phytophthora infestans*. Acta Sci Pol Hortorum Cultus.

[46] Goryluk A, Piórek M, Rekosz-Burlaga H, Studnicki M, Blaszczyk M (2016). Identification and bioactive properties of bacterial endophytes isolated from selected European herbal plants. Polish J Microbiol..

[47] Pawlik M, Cania B, Thijs S, Vangronsveld J, Piotrowska-Seget Z (2017). Hydrocarbon degradation potential and plant growth-promoting activity of culturable endophytic bacteria of *Lotus corniculatus* and *Oenothera biennis* from a long-term polluted site. Environ Sci Pollut Res Inter.

[48] Phillips LA, Germida JJ, Farrell RE, Greer CW (2008). Hydrocarbon degradation potential and activity of endophytic bacteria associated with prairie plants. Soil Biol Biochem..

[49] Baoune H, Ould EH, Pucci G, Sineli P, Loucif L, Polti MA (2018). Petroleum degradation by endophytic *Streptomyces* spp isolated from plants grown in contaminated soil of southern Algeria. Ecotoxicol Environ Safety..

[50] Khan S, Afzal M, Iqbal S, Khan QM (2013). Plant–bacteria partnerships for the remediation of hydrocarbon contaminated soils. Chemosphere.

[51] Ren JH, Li H, Wang JF, Ye JR, Yan AQ, Wu XQ (2013). Biocontrol potential of an endophytic *Bacillus pumilus* JK-SX001 against poplar canker. Biol. Contr..

[52] Jeong H, Choi SK, Kloepper JW, Ryu CM (2014). Genome sequence of the plant endophyte *Bacillus pumilus* INR7. Triggering induced systemic resistance in field crops. Genome Announc..

[53] Kumar A, Singh R, Yadav AD, Giri PK, Singh PK, Pandey KD (2016). Isolation and characterization of bacterial endophytes of *Curcuma longa* L. 3 Biotech..

[54] Buzanello EB, Rezende RP, Sousa FMO, Marques ELM, Loguercio LL (2014). A novel *Bacillus pumilus*-related strain from tropical landfarm soil is capable of rapid dibenzothiophene degradation and biodesulfurization. BMC Microbiol.

[55] Surendra SV, Mahalingam BL, Velan M (2017). Degradation of monoaromatics by *Bacillus pumilus* MVSV3. Braz Arch Biol Technol..

